# Effects of Roughage Combinations of Sugarcane Dry Leaves and Peanut Vine on the Rumen Microbial Community and Metabolites of Weaned Buffalo Calves

**DOI:** 10.3390/microorganisms14051050

**Published:** 2026-05-07

**Authors:** Caixiang Wei, Xin Gao, Ruizhanghui Wang, Qi Yan, Qichao Gu, Yuyang Liang, Dongwen Qiu, Yongqi Tan, Huadong Luo, Qingfeng Tang, Zhilin Yan, Jianwei Chen, Caixia Zou

**Affiliations:** 1 College of Animal Science and Technology, Guangxi University, Nanning 530005, China; 2218301034@st.gxu.edu.cn (C.W.); gaoxin961022@163.com (X.G.); wrzh0703@163.com (R.W.); 2318401013@st.gxu.edu.cn (Q.Y.); guqchao@gmail.com (Q.G.); a228191@163.com (Y.L.); 13768870337@163.com (D.Q.); renwoliu123@163.com (Y.T.); 13558173306@163.com (H.L.); 899qinghui@163.com (Q.T.); 15576333052@163.com (Z.Y.); 2134110220@st.gxu.edu.cn (J.C.); 2 Guangxi Key Laboratory of Animal Breeding, Disease Control and Prevention, Nanning 530004, China; 3 Science and Technology Backyard of Guangxi Fusui Dairy Industry, Chongzuo 532100, China

**Keywords:** complete pelleted diets, metabolites, rumen microbiota, sugarcane dry leaves, weaned buffalo calves

## Abstract

Based on previous findings that fiber digestibility and rumen fermentation in weaned buffalo calves were improved by a roughage combination of dried sugarcane leaves (SDL) and peanut vine (PV), this study reveals that the mechanism for improving fiber digestibility and growth performance involves increasing Succiniclasticum abundance and 3-methoxytyramine-betaxanthin level, which consequently increases ruminal acetate and propionate. Twenty-one calves were fed pelleted diets with roughage combinations the 15% SDL combined with PV (S15PV, 15% SDL + 15% PV), the 22.5% SDL combined with PV (S22.5PV, 22.5% SDL + 7.5% PV), or the 30% SDL combined with PV (S30PV, 30% SDL) for 63 days. The results showed no significant differences in α-diversity and β-diversity among the three groups (*p* > 0.05). A significantly higher relative abundance of *Succiniclasticum* was observed in the S22.5PV group than in the other two groups, by 241.57% and 136.25%, respectively (*p* < 0.05), and its effects were primarily exerted through carbohydrate and amino acid metabolism pathways. Differential metabolites were mainly enriched in cofactor/vitamin metabolism (vitamin B6, riboflavin) and amino acid pathways (arginine, tryptophan). By PLS-DA analysis, significantly higher levels of Bentyl and 3-Methoxytyramine-betaxanthin were observed in the S22.5PV group compared to the S15PV and S30PV groups, respectively. Positive correlations were observed between *Succiniclasticum* and NDFD, ADFD, acetic acid, propionic acid, isovaleric acid, as well as 3-Methoxytyramine-betaxanthin (*p* < 0.05). In conclusion, the rumen microbial diversity was not altered by the roughage combinations of dried sugarcane leaves and peanut vine, but the abundance of *Succiniclasticum* and the level of 3-Methoxytyramine-betaxanthin were significantly correlated with NDFD and ADFD, which enriched ruminal AA and PA, and may thus be associated with improved growth performance.

## 1. Introduction

Globally, with increasing emphasis on resource recycling and the prominent conflict of “competition for grain between humans and livestock,” developing economical and sustainable feed resources has become a critical issue for the livestock industry [1]. According to the Food and Agriculture Organization of the United Nations (FAO) statistics, from 2000 to 2022, global production of major crops (including cereals, sugarcane, vegetables, etc.) reached 9.6 billion tons, an increase of 56%. Sugarcane accounted for the highest proportion, approximately 20% (1.9 billion tons), followed by maize at 12% (1.2 billion tons) [2]. With continued growth in agricultural output, the annual production of various crop residues is projected to rise to about 200 billion tons [3], offering immense potential for utilizing renewable biological resources. Sugarcane is a crucial crop and a strategic reserve for sugar security in China [4]. As the world’s third-largest sugarcane producer, China’s annual output is about 100 million tons, holding a significant position in the global sugarcane industry [5].

Sugarcane (*Saccharum officinarum* L.) leaves, a by-product discarded in fields during harvest, consist of green leaves (fresh) and dry leaves (senescent or cleaned yellow leaves) (SDL). However, due to poor palatability, low digestibility, and high fiber content, a significant amount of dry leaves remains underutilized, often disposed of by burning, causing environmental pollution. Furthermore, weaned buffalo calves are at a critical stage of rumen development and microbial community establishment [6], with their ability to digest complex carbohydrates like cellulose progressively enhancing [7]. Studies show that moderate fiber supplementation promotes digestive tract development in calves, accelerates rumen papillae keratinization, and increases rumen volume and motility [6], and stimulates rumen development by enhancing substance exchange between rumen contents and blood, thereby improving the physiological phenotype [8]. However, due to the immature rumen of calves, their capacity to digest high-fiber materials is limited. Therefore, pelleting high-fiber ingredients can improve palatability and digestion [9,10].

Based on this, the effects of different roughage combinations of SDL and peanut vine (PV) (with SDL proportions of 15%, 22.5%, and 30%) on growth performance, nutrient apparent digestibility, and ruminal fermentation parameters in weaned buffalo calves were initially compared [11]. It was found that at the combination with 22.5% SDL and 7.5% PV, average daily gain (ADG), Acid detergent fiber (ADF) digestibility, Neutral detergent fiber (NDF) digestibility, and key ruminal nutrients such as Acetate acid (AA), Propionate acid (PA), and NH_3_-N were higher than in other treatment groups. The improvement in ADF and NDF digestibility suggests that a roughage combination containing a certain proportion of SDL has the potential to enhance rumen development [11]. However, the pathways through which this combination increases these key nutrients remain unclear. To investigate these mechanisms, this study integrated 16S rRNA sequencing and liquid chromatography-mass spectrometry (LC-MS) untargeted metabolomics technologies, focusing on the effects of different roughage combinations (especially the one with 22.5% SDL and 7.5% PV) on key rumen microbes like *Succiniclasticum* and metabolites such as 3-Methoxytyramine-betaxanthin, thereby revealing that the mechanism in improving fiber digestibility and growth performance involves increasing *Succiniclasticum* abundance and 3-methoxytyramine-betaxanthin level, which consequently increase ruminal acetate and propionate.

## 2. Materials and Methods

This study was approved by the Experimental Animal Management Committee of Guangxi University (Approval No.: GXU-2024-106).

### 2.1. Experimental Site and Time

The experiment was conducted from May to September 2024 at the breeding base of Royal Cell Biological Technology (Guangxi) Co., Ltd., located in Jiangxi Town, Jiangnan District, Nanning City, Guangxi (108.099° E, 22.775° N; subtropical monsoon climate), where the daily average temperature was approximately 22 °C to 35 °C.

### 2.2. Experimental Design

A single-factor completely randomized design was employed. Twenty-one healthy weaned female buffalo calves, aged (129.86 ± 7.82) days with a body weight of (67.38 ± 2.77) kg, were randomly divided into three groups, each housed in two pens with 3 and 4 calves per pen, respectively. The three groups were fed complete pelleted diets containing different proportions of sugarcane dry leaves (SDL) and peanut vine (PV): the 15% SDL combined with PV (S15PV) group received 15% SDL and 15% PV, the 22.5% SDL combined with PV (S22.5PV) group received 22.5% SDL and 7.5% PV, and the 30% SDL combined with PV (S30PV) group received 30% SDL and 0% PV. The experimental diets were formulated according to the “Feeding Standard of Beef Cattle” (NY/T 815-2004), with specific composition and nutritional levels detailed in Table 1. The trial lasted 73 days, including a 10-day adaptation period and a 63-day formal experimental period.

### 2.3. Feeding Management

The three experimental groups were housed in separate pens with identical facilities and environmental conditions. They were fed twice daily (08:00 and 15:00). Ad libitum access to feed and water was provided throughout the trial, and immunization and disinfection procedures followed company regulations. However, the digestibility trial and rumen fluid collection were conducted during the last week of the experiment, when each calf was moved to an individual pen for single−pen feeding., ensuring that all samples and measurements were collected from single-housed animals.

### 2.4. Measured Indices and Methods

The growth performance, nutrient digestibility, and rumen fermentation data of weaned buffalo calves were obtained from our published work [11]. To further explore their impact on host phenotypes, 16S rRNA sequencing and LC-MS untargeted metabolomics were performed on the same samples as described below.

#### 2.4.1. High-Throughput Sequencing Analysis of 16S rRNA Gene

Fresh rumen fluid samples (2 mL) were immediately immersed in liquid nitrogen for rapid freezing after collection, transferred to the laboratory within 2 h, and stored long-term in a −80 °C ultra-low temperature freezer (DW-HL528G, Zhongke Meiling Cryogenic Technology Co., Ltd., Hefei, China) for subsequent microbiological analysis. Total DNA was extracted from the samples using the E.Z.N.A.^®^ Soil DNA Kit (Omega Bio-tek, Norcross, GA, USA). DNA purity and concentration were measured using a NanoDrop 2000 spectrophotometer, and genomic DNA integrity was assessed via 1% agarose gel electrophoresis. Barcoded, region-specific primers were synthesized for the designated sequencing region, and PCR amplification was performed using TransGen AP221-02 TransStart Fastpfu DNA Polymerase on an ABI GeneAmp^®^ 9700 thermal cycler. After the amplification products were verified, PCR products from the same sample were pooled and analyzed by 2% agarose gel electrophoresis. The PCR products were then quantified using the QuantiFluor™-ST blue fluorescence quantification system (Promega Corporation, Madison, WI, USA). Following adapter ligation, self-ligated fragments were removed using magnetic bead purification. The library template was then enriched via PCR amplification, and the PCR products were recovered using magnetic beads. Finally, a PE250 library was constructed, and high-throughput sequencing was performed.

#### 2.4.2. LC/MS-Based Metabonomic Determination

Meanwhile, untargeted metabolomics analysis was performed on the same rumen fluid samples using liquid chromatography-mass spectrometry (LC-MS). The specific steps are as follows.

For untargeted metabolomic analysis, metabolite extraction commenced with a 100 µL aliquot of the liquid sample. This aliquot was mixed with pre-chilled 80% methanol via vigorous vortexing. The subsequent protocol followed the standardized procedure for tissue samples: incubation on ice for 5 min, followed by centrifugation at 15,000× *g* and 4 °C for 20 min. The resulting supernatant was then diluted with LC-MS grade water to a final methanol concentration of 53%. After a second centrifugation under identical conditions (15,000× *g*, 4 °C, 20 min), the clarified supernatant was collected and subjected to LC-MS/MS analysis [12,13].

1. UHPLC-MS/MS Analysis

Chromatographic separation was performed on a Vanquish UHPLC system (ThermoFisher Scientific, Dreieich, Germany) coupled to either an Orbitrap Q Exactive™ HF or an Orbitrap Q Exactive™ HF-X mass spectrometer (Thermo Fisher, Germany) at Novogene Co., Ltd. (Beijing, China). Samples were injected onto a Hypersil Gold column (100 × 2.1 mm, 1.9 µm) maintained at a constant flow rate of 0.2 mL/min. A 12 min linear gradient was employed using eluent A (0.1% formic acid in water) and eluent B (methanol). The gradient program was set as follows: 2% B held for 1.5 min, increased from 2% to 85% B over 3 min, further raised to 100% B over 10 min, returned to 2% B at 10.1 min, and re-equilibrated at 2% B until 12 min. The Q Exactive™ HF mass spectrometer was operated in positive/negative polarity switching mode with the following key parameters: spray voltage of 3.5 kV, capillary temperature of 320 °C, sheath gas flow rate of 35 psi, auxiliary gas flow rate of 10 L/min, S-lens RF level of 60, and auxiliary gas heater temperature of 350 °C.

2. Data processing and metabolite identification

Raw data files from UHPLC-MS/MS were processed using the XCMS package for peak detection [14], alignment, and quantification. Metabolite identification was achieved by matching the acquired MS/MS spectra against a high-quality reference spectral library, allowing for a mass tolerance of 10 ppm and considering common adduct ions. Background noise was subtracted by filtering signals present in blank samples. The raw quantitation data were normalized using the following formula to obtain relative peak areas: Relative peak area = (Raw sample quantitation value)/(Sum of all sample quantitation values/Sum of QC1 quantitation value). To ensure data quality, metabolites exhibiting a coefficient of variation (CV) greater than 30% across quality control (QC) samples were removed. All data processing was conducted on a Linux operating system (CentOS version 6.6) utilizing scripts written in R (v4.0.0) [15] and Python (v 3.9; Python Software Foundation, https://www.python.org (accessed on 25 September 2025)).

### 2.5. Data Analysis

#### 2.5.1. 16S rRNA Gene Sequencing

After obtaining raw data through high-throughput sequencing, barcode filtering, quality control, sequence assembly, and chimera removal were performed sequentially to obtain high-quality sequences. To maintain consistency with previous ruminant microbiome studies and to reduce the potential over-splitting that may arise from amplicon sequence variant (ASV)-based methods in low-abundance communities, an operational taxonomic unit (OTU)-based approach was adopted in this study. High-quality sequences were clustered into OTUs at 97% similarity, and species annotation was performed by alignment with reference databases to obtain representative OTU sequences and taxonomic information at various levels. A phylogenetic tree was also constructed to infer evolutionary relationships among species. To ensure comparability in α-diversity and β-diversity analyses, all samples were rarefied to normalize to the sequencing depth of the sample with the fewest sequences. Based on the OTU level, α-diversity indices, including Chao1, ACE, Shannon, and Simpson, were calculated using QIIME (v1.9.1) to evaluate bacterial community richness and diversity [16], and rarefaction curves were plotted to assess the overall saturation of the microbial communities. After obtaining relative abundance data, dimensionality reduction methods such as principal component analysis (PCA) were employed to assist in revealing differences and similarities in community structure among samples. Venn diagrams were used to display shared and unique species composition across groups. Differences in microbial community composition among groups were determined using the Kruskal–Wallis rank-sum test (p<0.05).

#### 2.5.2. Untargeted Metabolomics

Metabolites were annotated using the KEGG (https://www.genome.jp/kegg/pathway.html (accessed on 25 September 2025)), HMDB (https://hmdb.ca/metabolites (accessed on 25 September 2025)), and LIPIDMAPS databases (http://www.lipidmaps.org/ (accessed on 25 September 2025)). Multivariate statistical analyses, including principal component analysis (PCA) and partial least squares-discriminant analysis (PLS-DA), were conducted with the metaX software suite [17]. Univariate analysis (Student’s *t*-test) was applied to calculate the statistical significance (*p*-value) for each metabolite. Differential metabolites were identified based on the following criteria: a variable importance in projection (VIP) score from the PLS-DA model > 1, *p*-value < 0.05, and an absolute fold change (FC) ≥ 2 or FC ≤ 0.5.

Volcano plots were generated using the ggplot2 package (v3.5.1) in R to visualize the distribution of all metabolites based on their log_2_(FoldChange) and −log_10_(*p*-value), facilitating the graphical filtering of metabolites of interest. For hierarchical clustering analysis, the intensity areas of the differential metabolites were normalized to z-scores and presented in a heatmap created with the Pheatmap package (v1.0.13) in R.

Pairwise correlations between differential metabolites were calculated using Pearson’s method via the cor() function in R. The statistical significance of these correlations was determined with cor.mtest(), and significant correlations (*p*-value < 0.05) were visualized in a correlation plot constructed with the corrplot package (v0.95). Finally, to interpret the biological relevance, the functional roles and associated metabolic pathways of the differential metabolites were investigated through enrichment analysis based on the KEGG database. A metabolic pathway was considered significantly enriched when the ratio of differential metabolites mapped to a given pathway (x/n) exceeded the ratio of all identified metabolites mapped to that pathway (y/N), and the enrichment *p*-value < 0.05.

#### 2.5.3. Correlation Analysis

Spearman’s rank correlation analysis was conducted to assess the relationships among rumen fermentation parameters, nutrient apparent digestibility, significantly different microbial taxa (identified by Kruskal–Wallis rank-sum test, *p* < 0.05), and differential metabolites (VIP > 1 and *p* < 0.05) across all experimental samples. A heatmap of the resulting correlation matrix was generated using the pheatmap package (v1.0.13) in R.

#### 2.5.4. Statistical Analysis

Differences in rumen microbiota among the three groups were compared using the Kruskal–Wallis test followed by Dunn’s post hoc test with Bonferroni correction (*p* < 0.05) using SPSS 20.0. All data were collected from individually housed calves during the digestibility trial and rumen fluid collection; therefore, each calf was considered an independent experimental unit.

## 3. Results

### 3.1. Effects of Different Roughage Combinations on Rumen Microbial Diversity in Weaned Buffalo Calves

**Figure 1:**
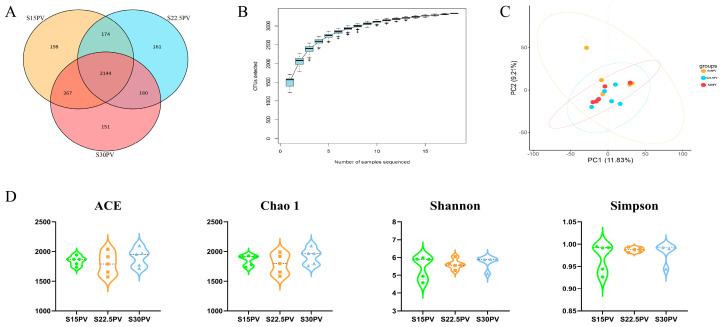
Effects of Different Roughage Combinations on the rumen microbiota of weaned buffalo calves. (**A**) Venn diagram showing operational taxonomic units (OTUs) among the three groups. (**B**) Rarefaction curves. (**C**) Principal component analysis (PCA). (**D**) α-diversity analysis. Rumen fluid samples were randomly collected from five calves per experimental group (*n* = 5). the 15% SDL combined with PV (S15PV); the 22.5% SDL combined with PV (S22.5PV); the 30% SDL combined with PV (S30PV). Green represents the S15PV group, orange represents the S22.5PV group, and blue represents the S30PV group.

To investigate the effects of replacing peanut vine with varying proportions of SDL on the rumen microbial community, 16S amplicon sequencing analysis was performed on 15 rumen content samples. A total of 2654 OTUs were identified across all samples, among which the S15PV, S22.5PV, and S30PV groups contained 198, 161, and 151 unique OTUs, respectively (Figure 1A). The OTU rarefaction curves for each group reached a plateau as the number of sequenced reads increased, indicating that the current sequencing depth and sample size were sufficient to cover the major composition of the rumen microbial community (Figure 1B).

Further principal component analysis (PCA) revealed no significant differences in microbial community structure among the S15PV, S22.5PV, and S30PV groups (*p* > 0.05) (Figure 1C). α-diversity analysis showed that the ACE, Chao1, Simpson, and Shannon indices did not differ significantly among the three groups (*p* > 0.05) (Figure 1D). Collectively, these results indicate that under the conditions of this study, different roughage combinations (with up to 30% SDL) did not significantly affect rumen microbial diversity, community structure, or major taxonomic composition.

### 3.2. Effects of Different Roughage Combinations on Rumen Microbial Community Structure in Weaned Buffalo Calves

**Figure 2:**
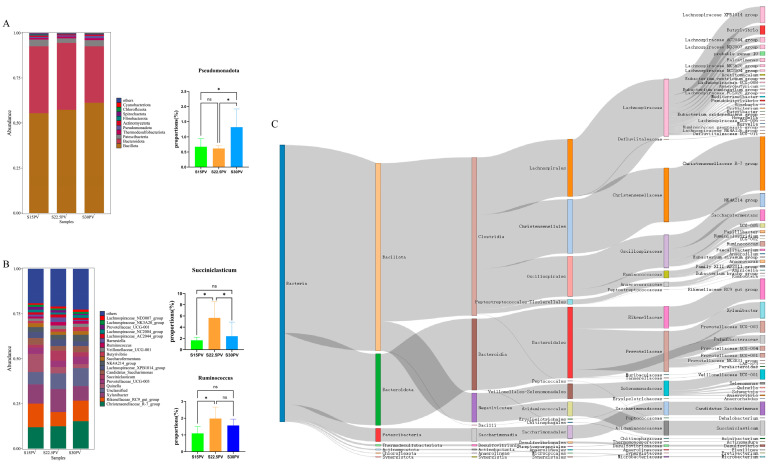
Effects of Different Roughage Combinations on the rumen microbial community structure of weaned buffalo calves. The asterisk (*) indicates a significant difference (*p* < 0.05), and the same applies to the figure below, ns indicates No Significance. (**A**) Relative abundance of microbial communities at the phylum level. (*p* < 0.05). (**B**) Relative abundance of microbial communities at the genus level. (**C**) Sankey diagram showing phylum-to-genus taxonomic relationships of rumen bacteria. Rumen fluid samples were randomly collected from five calves per experimental group (*n* = 5).

Analysis of the rumen bacterial community composition revealed that at the phylum level, the top five phyla were *Bacillota* (ranging from 55.39% to 61.17%), *Bacteroidota* (ranging from 31.43% to 37.22%), *Patescibacteria* (ranging from 2.54% to 3.75%), *Thermodesulfobacteriota* (ranging from 0.86% to 1.04%), and *Pseudomonadota* (ranging from 0.62% to 1.38%). The Kruskal–Wallis test was performed on the top ten differentially abundant phyla, revealing that the relative abundance of *Pseudomonadota* in the S30PV group was significantly higher than that in the other two groups (*p* < 0.05) (Figure 2A). At the genus level, the top five genera were the *Christensenellaceae R-7 group* (ranging from 11.91% to 15.27%), *Rikenellaceae RC9 gut group* (ranging from 7.89% to 13.12%), *Xylanibacter* (ranging from 8.08% to 12.80%), *Quinella* (ranging from 2.20% to 10.74%), and *Succiniclasticum* (ranging from 1.66% to 5.67%). The Kruskal–Wallis test on the top twenty differentially abundant genera revealed that the relative abundance of *Succiniclasticum* in the S22.5PV group (mean 5.67%, median 4.45%, IQR 4.88%) was significantly higher than that in the S15PV group (mean 1.66%, median 1.46%, IQR 0.99%) and the S30PV group (mean 2.40%, median 1.36%, IQR 3.36%) (*p* < 0.05). Moreover, the relative abundance of *Ruminococcus* in the S22.5PV group was significantly higher than that in the S15PV group (*p* < 0.05) (Figure 2B). To trace the taxonomic origin of the differentially abundant genus *Succiniclasticum*, a Sankey diagram was constructed from the phylum to genus level (Figure 2C). The analysis revealed that *Succiniclasticum* originated exclusively from the family *Acidaminococcaceae* (relative abundance 2.51%), which further traced back to *Acidaminococcales* (2.51%), *Negativicutes* (5.08%), and ultimately to the phylum *Bacillota* (32.67%). Notably, the flow from *Negativicutes* to *Acidaminococcales* accounted for 49.3% of the total relative abundance, and *Succiniclasticum* constituted the entire abundance of *Acidaminococcaceae* in this dataset. These results indicate that *Succiniclasticum* is a representative member of the phylum *Bacillota* within the rumen microbiota, and its significant intergroup differences may reflect an overall compositional shift within this phylum.

### 3.3. Effects of Different Roughage Combinations on KEGG Annotation of Rumen Microbiota in Weaned Buffalo Calves

**Figure 3:**
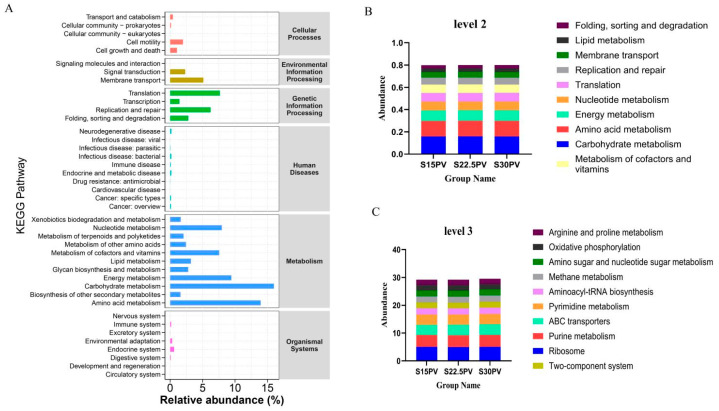
KEGG functional prediction analysis of rumen microbiota in weaned buffalo calves. (**A**) KEGG pathway level 1 functional classification of rumen microbiota. (**B**) KEGG pathway level 2 functional classification of rumen microbiota. (**C**) KEGG pathway level 3 functional classification of rumen microbiota. Rumen fluid samples were randomly collected from five calves per experimental group (*n* = 5).

To further elucidate the metabolic potential of the rumen microbial community, KEGG functional prediction analysis was performed based on 16S rRNA gene sequencing data. At KEGG pathway level 1, Metabolism was the most abundant functional category, accounting for 68.1–70.5% of the total functional annotations (Figure 3A). Analysis of KEGG level 2 pathways revealed that Carbohydrate metabolism was the most abundant pathway across the three groups (15.4–16.5%), followed by Amino acid metabolism (13.6–14.2%); intergroup comparisons showed no significant differences in either Carbohydrate metabolism or Amino acid metabolism (*p* > 0.05) (Figure 3B). Further analysis at KEGG level 3 pathways revealed that the most abundant pathways across the three groups were Ribosome (4.9–5.3%), followed by Purine metabolism (4.1–4.5%), and ABC transporters (2.7–4.4%). In summary, pelleted diets with different roughage combinations may primarily influence the expression of genes associated with Ribosome, Purine metabolism, and ABC transporters through the regulation of Carbohydrate metabolism and Amino acid metabolism pathways.

### 3.4. Effects of Different Roughage Combinations on Rumen Microbial Metabolites in Weaned Buffalo Calves

**Figure 4:**
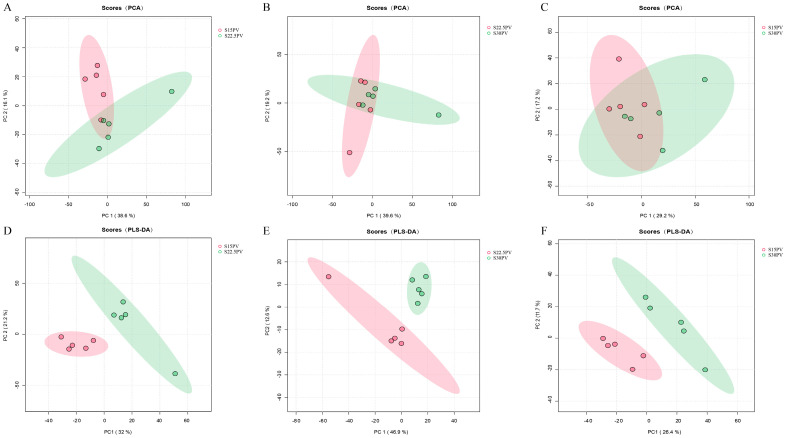
Principal component analysis (PCA) and partial least squares-discriminant analysis (PLS-DA) score plots. (**A**–**C**) PCA score plots comparing S15PV vs. S22.5PV, S22.5PV vs. S30PV, and S15PV vs. S30PV groups in positive and negative ion modes, respectively. (**D**–**F**) PLS-DA score plots comparing S15PV vs. S22.5PV, S22.5PV vs. S30PV, and S15PV vs. S30PV groups in positive and negative ion modes, respectively. Rumen fluid samples were randomly collected from five calves per experimental group (*n* = 5).

A bidirectional regulatory relationship exists between rumen microbes and metabolites. Therefore, untargeted metabolomics was further employed to investigate the effects of SDL substitution for PV at different ratios on rumen microbial metabolites in weaned buffalo calves. We identified 1334 and 1030 metabolites in positive and negative ion modes, respectively, and differential metabolites were further screened using the criteria of VIP > 1, *p* < 0.05, and |FC| ≥ 2 or ≤0.5 (Table S4). PCA and PLS-DA were used to assess inter-group differences, where greater spatial separation in score plots indicates more significant metabolic differences. While PCA did not show significant separation among the three groups, PLS-DA score plots revealed clear separation between all groups, suggesting the existence of some metabolic differences (Figure 4A–F).

**Figure 5:**
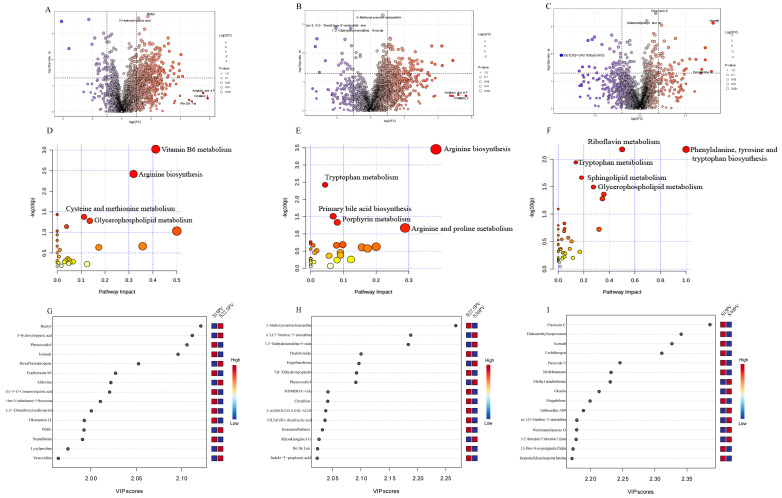
Differential analysis of rumen microbiota metabolites among different groups. (**A**–**C**) Differential metabolite volcano plots in positive and negative ion modes for the S15PV vs. S22.5PV, S22.5PV vs. S30PV, and S15PV vs. S30PV group comparisons. (**D**–**F**) Metabolite pathway enrichment analysis for the S15PV vs. S22.5PV, S22.5PV vs. S30PV, and S15PV vs. S30PV group comparisons. The horizontal axis represents pathway impact, and the vertical axis represents pathway enrichment. The size of the circle corresponds to the degree of pathway enrichment, with larger circles indicating greater enrichment. Darker colors indicate greater impact. (**G**–**I**) Top 15 metabolites with the strongest predictive accuracy in PLS-DA analysis for the S15PV vs. S22.5PV, S22.5PV vs. S30PV, and S15PV vs. S30PV group comparisons, ranked by importance (from top to bottom). Colored squares on the right represent the relative levels of the corresponding metabolites in each group.

Following LC-MS-based analysis, significant differential metabolites were identified according to the criteria of VIP value >1 and *p* < 0.05 (Figure 5A–C). The Table S4 presents comprehensive differential metabolite data under positive and negative ion modes for the comparisons between the S15PV and S22.5PV groups, the S22.5PV and S30PV groups, and the S15PV and S30PV groups. The results showed that 372 differential metabolites, including Bentyl and 3-Hydroxyhippuric acid, were identified between the S15PV and S22.5PV groups (Figure 5A). Between the S22.5PV and S30PV groups, 252 differential metabolites, including 3-Methoxytyramine-betaxanthin and cis-3,4′,5-Trimethoxy-3′-aminostilbene, were identified (Figure 5B). Between the S15PV and S30PV groups, 137 differential metabolites, including Piscrocin E and Didesmethylisoproturon, were identified (Figure 5C). The differential metabolites were mainly classified into categories such as organic acids and their derivatives, lipids and lipid-like molecules, organoheterocyclic compounds, and benzenoids (Table S5), with organic acids and their derivatives being the largest category. Next, based on the Kyoto Encyclopedia of Genes and Genomes (KEGG) pathway analysis, the differential metabolites were analyzed for the three group comparisons, revealing that they were primarily enriched in various metabolic pathways, with the most prominent pathways being associated with cofactor and vitamin metabolism, such as vitamin B6 metabolism and riboflavin metabolism, as well as amino acid metabolism pathways, including arginine metabolism, tryptophan metabolism, and phenylalanine, tyrosine, and tryptophan biosynthesis (Figure 5D–F). In addition, PLS-DA prediction model analysis was performed using metabolite levels as variables, and the results further indicated that Bentyl was the most significant metabolite in the S15PV vs. S22.5PV comparison (Figure 5G), 3-Methoxytyramine-betaxanthin was the most significant metabolite in the S22.5PV vs. S30PV comparison (Figure 5H), and Piscrocin E was the most significant metabolite in the S15PV vs. S30PV comparison (Figure 5I). In summary, different roughage combinations induced significant changes in metabolite composition, and among them, the combination with 22.5% SDL and 7.5% PV showed the most prominent effects in regulating key metabolites.

### 3.5. Correlation Analysis Among Nutrient Apparent Digestibility, Rumen Fermentation Parameters, Differential Microbes, and Differential Metabolites

**Figure 6:**
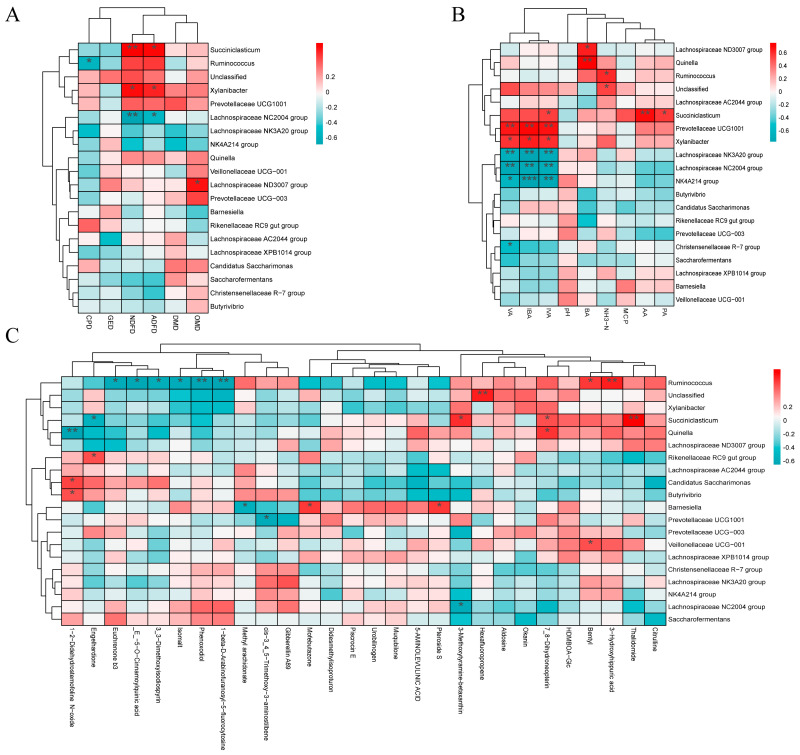
Spearman correlation analysis. (**A**) Correlation analysis between apparent nutrient digestibility and the top 20 differential microbes at the genus level. (**B**) Correlation analysis between rumen fermentation parameters and the top 20 differential microbes at the genus level. (**C**) Correlation analysis between the top 20 differentially abundant microbial genera and the top 10 differential metabolites across the three pairwise comparisons (S15PV vs. S22.5PV, S22.5PV vs. S30PV, and S15PV vs. S30PV). Red indicates positive correlation, blue indicates negative correlation, and darker colors represent stronger correlations. * *p* < 0.05 indicates a significant difference, ** *p* < 0.01 indicates a very significant difference, *** *p* < 0.001 indicates a highly significant difference.

To explore the potential relationships among nutrient apparent digestibility, rumen fermentation parameters, microbial community, and metabolites in weaned buffalo calves fed complete pelleted diets with SDL substituting for PV at different ratios, we performed Spearman correlation analysis. First, the correlation between nutrient apparent digestibility and the top 20 differentially abundant microbial species was analyzed, and the results showed that *Succiniclasticum* was significantly positively correlated with NDF digestibility and ADF digestibility (*p* < 0.05) (Figure 6A). Furthermore, the correlation between rumen fermentation parameters and the top 20 differentially abundant microbial species was analyzed, revealing that *Succiniclasticum* was significantly positively correlated with AA, PA, and IVA (*p* < 0.05), while *Ruminococcus* was significantly positively correlated with NH_3_-N (*p* < 0.05) (Figure 6B). Additionally, the correlation between the top 20 differentially abundant microbial species and the top 10 differential metabolites among the three groups was analyzed, showing that *Succiniclasticum* was significantly positively correlated with 3-Methoxytyramine-betaxanthin and Thalidomide, and *Ruminococcus* was significantly positively correlated with Bentyl and 3-Hydroxyhippuric acid (Figure 6C). In summary, network-like correlations exist among differentially abundant microbial species, differential metabolites, rumen fermentation parameters, and nutrient apparent digestibility, suggesting that different roughage combinations may regulate host nutrient metabolism by influencing rumen microorganisms and their metabolites. Notably, the combination with 22.5% SDL and 7.5% PV exhibited the best regulatory effects through enrichment of *Succiniclasticum* and 3-Methoxytyramine-betaxanthin.

## 4. Discussion

### 4.1. Effects of Different Roughage Combinations Containing Dried Sugarcane Leaves on Rumen Microbial Community and Metabolic Pathways in Weaned Buffalo Calves

The rumen is a dynamic ecosystem inhabited by an extremely complex community of bacteria, archaea, fungi, and protozoa [18]. Structural carbohydrates in the diet are efficiently degraded synergistically by these microbes and are converted into volatile fatty acids (VFAs) and microbial protein [19]. VFAs are considered the primary energy source for ruminants, while crucial amino acids are provided by microbial protein [20]. Therefore, feed utilization efficiency, production performance, and health status of ruminants are directly determined by the community structure and function of rumen microbiota. α-diversity and β-diversity are included in rumen microbial diversity. It was noted by Henderson [21] that the composition of the rumen microbial community is influenced not only by external factors like diet but also is closely related to internal factors such as host individual differences and feeding habits. In this study, no significant differences in ruminal α/β-diversity were observed among the groups, which may be attributed to individual variation, the stability of the core microbial community, and the generally high crude protein content masking intergroup differences. However, roughage combinations with up to 30% SDL did not disrupt the microbial structure, indicating no adverse effects on ruminants.

At the phylum level, the rumen microbiota in ruminants is predominantly composed of *Bacillota* and *Bacteroidota* [22]. *Bacillota* can produce lipases, proteases, and cellulases, which decompose cellulose to generate short-chain fatty acids and lipids, thereby promoting body fat deposition [23]. *Bacteroidota* is involved in carbohydrate and protein metabolism, facilitating the absorption of lipids, proteins, and polysaccharides [24]. In this study, there were no significant differences in the relative abundances of *Bacillota* and *Bacteroidota* among the groups, indicating that the inclusion of up to 30% dried sugarcane leaves did not affect the overall structure of the rumen microbial community. However, the relative abundance of *Pseudomonadota* in the S30PV group was significantly higher than that in the other groups. Studies have shown that overgrowth of *Pseudomonadota* may lead to microbial dysbiosis, triggering intestinal inflammation and diarrhea [25], ultimately resulting in weight loss and reduced growth [26]. This is consistent with the previously reported trend in ADG, where the ADG of the S30PV group was lower than that of the S15PV and S22.5PV groups [11]. Further analysis at the genus level revealed that the relative abundance of *Succiniclasticum* in the S22.5PV group was significantly higher than that in the other groups, and the relative abundance of *Ruminococcus* was significantly higher than that in the S15PV group. *Succiniclasticum* is an important propionate-producing bacterium in the rumen that converts succinate to propionate via the succinate pathway, thereby improving fiber utilization and providing precursors for host gluconeogenesis [27]. Studies have shown that the enrichment of this genus is positively correlated with growth performance in ruminants; for example, the abundance of *Succiniclasticum* in the rumen of male lambs is significantly higher than that in females and is closely associated with body weight gain [28]. Additionally, high-fat diets can enrich *Succiniclasticum* and promote propionate-type fermentation while enhancing energy and nitrogen utilization efficiency [29], which is consistent with the previously observed trend in propionate (PA) levels (PA in the S22.5PV group was significantly higher than that in the S30PV group) [11], suggesting its key role in regulating energy metabolism. *Ruminococcus* is an important fibrolytic bacterium in the rumen that efficiently degrades cellulose to produce volatile fatty acids such as acetate and propionate. Its metabolism requires nitrogen sources for bacterial protein synthesis, and its enrichment reflects an overall enhancement of the rumen microbiota’s regulation of fiber degradation and nitrogen metabolism [30]. Sankey diagram tracing revealed that *Succiniclasticum* belongs to a lineage within the phylum *Bacillota*. Notably, although there was no significant difference in the relative abundance of *Bacillota* among the groups, the abundance of *Succiniclasticum*, a key functional genus within this phylum, differed significantly. This finding is consistent with the results reported by Li et al. [29] and reflects the functional redundancy and niche differentiation of the microbial community, indicating that while *Bacillota* as a whole maintains its ecological niche in the rumen, the members responsible for key metabolic functions such as propionate production undergo turnover.

Combined with the KEGG annotation analysis of the rumen microbiota, it was found that carbohydrate metabolism and amino acid metabolism were the two most predominant metabolic pathways. Among them, the activity of carbohydrate metabolism pathways is closely related to the type of carbohydrates in the diet [31]. For instance, non-structural carbohydrates such as starch and sugars are primarily metabolized through glycolysis and subsequent acrylate pathways, with propionate as the main end product [32,33]. In contrast, structural carbohydrates such as cellulose are mainly degraded by fibrolytic bacteria, releasing large amounts of H_2_, which in turn enriches hydrogenotrophic acetogenic bacteria that can utilize H_2_ and CO_2_ to synthesize acetate [34]. Therefore, this further suggests that *Succiniclasticum* may play a potential role in regulating key fermentation products such as acetate and propionate through its involvement in carbohydrate metabolism pathways.

In summary, the inclusion of up to 30% dried sugarcane leaves did not alter the core rumen microbiota. The higher levels of AA and PA observed in the S22.5PV group compared to the S30PV group may be attributed to the reduced relative abundance of *Pseudomonadota* and the increased relative abundance of *Succiniclasticum* in the S22.5PV group. *Succiniclasticum* generates propionate via carbohydrate metabolism pathways, thereby promoting gluconeogenesis and providing energy for the host, which in turn enhances ADG.

### 4.2. Effects of Different Roughage Combinations on Rumen Metabolites and Key Metabolic Pathways in Weaned Buffalo Calves

Untargeted metabolomics analysis revealed that organic acids and derivatives constituted the largest category of differential metabolites. KEGG pathway enrichment analysis showed that the arginine metabolism pathway was significantly enriched in both the S15PV vs. S22.5PV and S22.5PV vs. S30PV comparisons, but not in the S15PV vs. S30PV comparison. This discrepancy indicates that when the SDL proportion in the roughage combination is within 22.5%, the effect is primarily mediated through the arginine metabolism pathway. Arginine is a precursor amino acid for key substances such as urea and nitric oxide, and is involved in core metabolic processes related to animal health, growth, reproduction, and homeostasis [35]. It plays a critical role in nitrogen transport and storage [36,37]. The Nutrient Requirements of Dairy Cattle (NASEM, 2021) [38] also classifies arginine as one of the ten essential amino acids (EAA), which is crucial for the lactation and reproduction performance of high-yielding dairy cows. Notably, *Succiniclasticum* is closely associated with arginine metabolism. Genomic analyses have shown that most members of the phylum *Bacillota* synthesize arginine via succinylated intermediates [39]. Metabolomic studies further confirm that when *Succiniclasticum* is enriched, the arginine biosynthesis pathway in the rumen is significantly enriched [40]. This suggests that when the replacement of PV with SDL affects arginine levels in the rumen, the abundance or activity of *Succiniclasticum* may undergo adaptive changes, thereby influencing the efficiency of arginine metabolism within the microbial community. Combined with KEGG annotation analysis of the rumen microbiota, it was found that amino acid metabolism pathways primarily affect the expression of ABC transporter genes, which are involved in the transport of arginine and its derivatives [41]. Collectively, these results indicate that the increased relative abundance of *Succiniclasticum* may enhance arginine metabolism by enriching amino acid metabolism-related functions, particularly via the ABC transporter system, thereby increasing arginine availability and promoting ADG.

Combined with PLS-DA predictive analysis, it was further revealed that the most significant differential metabolites in the S15PV vs. S22.5PV and S22.5PV vs. S30PV comparisons were Bentyl and 3-Methoxytyramine-betaxanthin, respectively, both of which were significantly upregulated in the S22.5PV group. Notably, a synchronous increase in the abundance of 3-Methoxytyramine-betaxanthin and *Succiniclasticum* was observed in the S22.5PV group. Tyrosine is catalyzed by tyrosinase to produce L-DOPA, which is then decarboxylated to generate dopamine. Dopamine is methylated to 3-methoxytyramine (3-MT) by catechol-O-methyltransferase (COMT). On one hand, 3-Methoxytyramine-betaxanthin is formed from 3-MT via non-enzymatic spontaneous condensation [42]; on the other hand, 3-MT may be utilized by ruminal acetogenic bacteria to produce acetate through the reductive acetogenesis pathway [43]. It is suggested by this that the enrichment of this compound in the rumen may reflect enhanced acetogenic activity, thereby indirectly indicating an increased potential for acetate production. Furthermore, the enrichment of *Succiniclasticum* is closely associated with the tyrosine metabolism pathway in the rumen. It was found by Wang et al. [44] that *Succiniclasticum* was enriched and the tyrosine metabolism pathway was activated by a high-energy, high-protein diet in Cashmere goats. Similarly, it was reported by Wang et al. [28] in Hu sheep that the enrichment of *Succiniclasticum* in male lambs was accompanied by significant upregulation of the tyrosine metabolism pathway, and positive correlations were observed between tyrosine metabolites and body weight. Collectively, it is suggested by these results that the increased relative abundance of *Succiniclasticum* may enrich the tyrosine metabolism pathway, thereby promoting elevated concentrations of 3-Methoxytyramine-betaxanthin.

In summary, the roughage combination with 22.5% SDL and 7.5% PV increased the relative abundance of *Succiniclasticum*. On one hand, it may regulate the arginine metabolism pathway via the ABC transporter system, thereby increasing arginine concentration in the host. On the other hand, it may enrich the tyrosine metabolism pathway, promoting the production of 3-Methoxytyramine-betaxanthin while simultaneously increasing acetate content, collectively providing energy and metabolic precursors for the host.

### 4.3. Correlation Analysis Among Nutrient Digestibility, Rumen Fermentation Parameters, Key Fibrolytic Genera (Succiniclasticum and Ruminococcus), and Differential Metabolites in Weaned Buffalo Calves

It was shown by correlation analysis that *Succiniclasticum* was significantly positively correlated with NDFD, ADFD, AA, PA, and IVA, which is consistent with the findings of Guan et al., [45] that enrichment of the genus *Succiniclasticum* increases the diversity and abundance of cellulases and hemicellulases, and serves as a key genus involved in the VFA pathway. This further corroborates that this genus may convert succinate to propionate via the succinate pathway, thereby improving fiber utilization. Further analysis of the correlation between key microorganisms and key metabolites revealed that *Succiniclasticum* was significantly positively correlated with 3-Methoxytyramine-betaxanthin, 7,8-Dihydroneopterin, and Thalidomide. Notably, PLS-DA predictive analysis showed that in the S22.5PV vs. S30PV comparison, both 3-Methoxytyramine-betaxanthin and Thalidomide were significantly enriched in the S22.5PV group, further suggesting that the roughage combination with 22.5% SDL and 7.5% PV may indirectly promote the enrichment of the above metabolites by increasing the relative abundance of *Succiniclasticum*. In addition, we found that *Ruminococcus* was significantly positively correlated with Bentyl and 3-Hydroxyhippuric acid. PLS-DA predictive analysis indicated that in the S15PV vs. S22.5PV comparison, both Bentyl and 3-Hydroxyhippuric acid were significantly enriched in the S22.5PV group, suggesting that the inclusion of 22.5% SDL and 7.5% PV may indirectly promote the production of these metabolites by increasing the relative abundance of *Ruminococcus*. Elevated levels of 3-Hydroxyhippuric acid are often accompanied by improved ruminal propionate concentration, which may be attributed to the activity of fiber-degrading bacteria promoting substrate breakdown and utilization, thereby enhancing nutrient utilization efficiency.

In summary, the roughage combination with 22.5% SDL and 7.5% PV increased the relative abundance of *Succiniclasticum* and *Ruminococcus*. The former may indirectly promote the production of 3-Methoxytyramine-betaxanthin, 7,8-Dihydroneopterin, and Thalidomide, thereby increasing the levels of NDFD, ADFD, AA, PA, and IVA; while *Ruminococcus* may indirectly promote the enrichment of Bentyl and 3-Hydroxyhippuric acid, reflecting the active metabolism of fibrolytic bacteria.

## 5. Conclusions

Overall, Pelleted diets containing a roughage combination of 22.5% SDL and 7.5% PV may enhance cellulose digestion and utilization, which may be mainly attributed to the increased abundance of the key fibrolytic genus Succiniclasticum, which in turn regulates the production of the key metabolite 3-Methoxytyramine-betaxanthin. This may synergistically enhance ruminal fiber digestion and ultimately increase key fermentation parameters (AA, PA) and nutrient digestibility (NDFD, ADFD) in the rumen, providing a nutritional foundation for improved growth performance. This study confirms that using SDL as a fiber source in pelleted weaning diets for buffalo calves is feasible.

## Figures and Tables

**Table 1:** Composition and nutritional level of complete pelleted diets using sugarcane dry leaves as a substitute for peanut vines with different proportions (DM, %).

Items	S15PV	S22.5PV	S30PV
Ingredients			
Corn	32.00	32.00	32.00
Soybean meal	30.40	30.40	30.40
Limestone	1.80	1.80	1.80
CaHPO_4_	1.20	1.20	1.20
NaCl	0.60	0.60	0.60
sugarcane dry leaves	15.00	22.50	30.00
Peanut vine	15.00	7.50	0.00
Premix ^(1)^	4.00	4.00	4.00
Total	100.00	100.00	100.00
Nutrient levels ^(2)^			
DM (air-dry basis)	94.43	92.85	92.10
NE_mf_/(MJ/kg)	10.45	10.34	10.13
CP	22.50	21.00	19.10
EE	2.60	2.58	2.56
NDF	35.49	37.23	41.37
ADF	13.38	14.94	16.32
Ca	0.83	0.95	1.12
P	0.62	0.55	0.49

^1^ The premix provided the following per kg of the premix: VA 5000 IU, VD_3_ 5000 IU, VE 60 IU, VB_1_ 147 mg, VB_12_ 0.1 mg, Cu (as copper sulfate) 12.5 mg, Fe (as ferrous sulfate) 90 mg, Zn (as zinc sulfate) 100 mg, Mn (as manganese sulfate) 60 mg, I (as potassium iodide) 0.7 mg, Se (as sodium selenite) 0.3 mg, Co (as Cobalt carbonate) 0.2 mg. ^2^ NE_mf_ was a calculated value according to Feeding Standard of *Beef Cattle* (NY/T 815-2004), and the other nutrient levels were measured values. Note: CaHPO_4_ is dicalcium phosphate; NaCl is salt; DM is dry matter; CP is crude protein; EE is ether extract; NDF is neutral detergent fiber; ADF is acid detergent fiber; Ca is calcium; P is phosphorus.

## Data Availability

The raw 16S rRNA gene sequencing data and untargeted metabolomics data generated in this study will be deposited in a public repository (NCBI SRA) upon acceptance or as required by the journal. The accession numbers will be provided during the revision process or before final publication.

## Supplementary Materials

The following supporting information can be downloaded at: https://www.mdpi.com/article/10.3390/microorganisms14051050/s1, Table S1: The effect of SDL replacing different proportions of PVs in complete pelleted diets on the growth performance of weaned calf water buffalo; Table S2: The effect of SDL replacing different proportions of PVs in complete pelleted diets on the apparent digestibility of nutrients in weaned calf water buffalo; Table S3: The effect of SDL replacing different proportions of PVs in complete pelleted diets on rumen fermentation parameters of weaned calf water buffalo; Table S4: The significantly different metabolites in positive (pos) and negative (neg) ion mode; Table S5: Classification of differential metabolite subclasses in positive (pos) and negative (neg) ion model.
